# Stakeholder input on a care planning tool to address unhealthy behaviors, mental health needs, and social risks: The value of different stakeholder perspectives

**DOI:** 10.1017/cts.2021.864

**Published:** 2021-10-07

**Authors:** Kristen O’Loughlin, Alison N. Huffstetler, Hannah Shadowen, E. Marshall Brooks, Jennifer Hinesley, Amy G. Huebschmann, Russell E. Glasgow, Arline Bohannon, Alex H. Krist

**Affiliations:** 1 Department of Family Medicine and Population Health, Virginia Commonwealth University, Richmond, VA, USA; 2 Department of Psychology, Virginia Commonwealth University, Richmond, VA, USA; 3 Department of Medicine, Division of General Internal Medicine, University of Colorado, Aurora, CO, USA; 4 Adult and Child Consortium for Outcomes Research and Delivery Science, University of Colorado, Aurora, CO, USA; 5 University of Colorado Anschutz Medical Campus, Aurora, CO, USA

**Keywords:** Chronic disease, chronic disease management, patient care planning, health technology, intervention design, stakeholder input, community-engaged research

## Abstract

This report describes how stakeholder groups informed a web-based care planning tool’s development for addressing root causes of poor health. Stakeholders included community members (n = 6), researchers (n = 6), community care providers (n = 9), and patients (n = 17). Feedback was solicited through focus groups, semi-structured interviews, and user experience observations and then qualitatively analyzed to identify themes. Each group contributed a unique perspective. Researchers wanted evidence-based content; community members and providers focused on making goals manageable; patients wanted care team support and simple action-oriented language. Our findings highlight the benefits of stakeholder input. Blending perspectives from multiple groups results in a more robust intervention design.

## Introduction

Patients with poorly controlled multiple chronic conditions (MCCs) often have unhealthy behaviors, poor mental health, and unmet social needs which can complicate outpatient management. One approach to addressing these root causes is for patients to create health-related goals and care plans and then get help from a care team to achieve their goals [1]. Patient-centered care planning first involves assessing patient health risks and identifying which needs to prioritize [2]. Patients then define a personal goal and select strategies for achieving it. Previous studies demonstrate that disease-specific care plans can improve management of conditions and quality of life [3,4].

Care planning can be difficult for patients to complete without guidance and is time-consuming for clinicians, but digital health tools can help facilitate this process [5]. We are thus expanding the previously tested web-based screening tool My Own Health Report (MOHR) [6], to support patients in creating care plans for health behaviors, mental health, and social needs. MOHR was developed to assess these risks, but did not include a formal care planning process. This expansion is a part of a larger trial testing a community strategy to address root causes of uncontrolled MCCs, which involves using patient navigators, community health workers, and clinical-community linkages to help patients achieve personal care plan goals.

Creating a care planning tool that is patient-centered, evidence-based, and promotes shared decision-making regarding multiple health risk factors is difficult. Authentic engagement of stakeholder groups can be used while creating a health intervention in order to enhance its adoption and implementation [7–9]. Recent guidance for researchers on best practices for health intervention development includes recommendations to engage relevant groups throughout the process [10]. However, stakeholders are often not included in intervention development processes and there is a lack of information about the engagement process and outcomes when stakeholders are included [11]. Therefore, there is a need for examples in the literature of researchers meaningfully engaging stakeholder groups and the impact created by their involvement.

We solicited feedback from four diverse stakeholder groups to inform the design, use, and impact of MOHR’s expansion. These groups included community members, academic researchers, community service professionals (CSP), and patients. This paper reports on the feedback received from each stakeholder group, how it shaped the tool’s final form, and implications for researchers doing similar work.

## Methods

Using a qualitative approach, this study sought and evaluated feedback from four stakeholder groups to inform the design of a web-based care planning tool. As summarized in Table 1, the research team engaged each group separately using a focus group, structured feedback, or semi-structured interviews. Throughout the process, stakeholders were asked to consider the MOHR care planning concept, content, understandability, feasibility, and/or usability. This study was approved by the university Institutional Review Board (IRB HM20015553) and conducted between November 2019 and August 2020.

**Table 1.** Major domains of stakeholder influence on my own health report care planning design

Stakeholder group	Engagement strategy	Domain of feedback
*Community Members* (*n* = 6): Residents of the East End of Richmond, an underserved urban community	In-person focus group	Whether the care planning process was feasible and helped patients
*Academic Researchers* (*n* = 6): Doctorally trained university professors trained in internal medicine, family medicine, and/or clinical psychology	Semi-structured review via email	Whether MOHR’s questions and strategies for care plans were evidence-based
*Community Care Providers* (*n* = 8): Healthcare professionals employed as community engagement coordinators, directors of community health and wellness programs, social workers, and dentists	Semi-structured review via email	Ideas on practical approaches for care plans and community resources
*Patients* (*n* = 17): Patients with multiple chronic conditions and/or social needs receiving care at a family medicine practice	In-person user experience observations and interviews	How to make the care planning process intuitive and useful and support needed from the care team to take action

### Community Member Engagement

Six community members (83% female) were recruited from an existing community partnership, Engaging Richmond, which serves the disadvantaged, urban, East End of Richmond, Virginia [9,12]. Feedback was solicited through a focus group facilitated by two members of the research team. Facilitators demonstrated the MOHR online tool content and prompted feedback on appearance, usability, goal creation workflow, strategies to achieve goals, and the roles of patient navigators and community health workers. The focus group was audio-recorded and transcribed.

### Academic Researcher and CSP Engagement

Six academic clinicians and researchers (33% female) participated who were involved in the larger study as content experts in chronic disease, behavior change, and/or community-oriented primary care. All had doctorate-level training in internal medicine, family medicine, or clinical psychology. Nine CSP (89% female) caring for patients’ health-related needs in the community were recruited. CSP participants included community engagement coordinators, directors of community health and wellness, social workers, and dentists. Feedback from both groups was collected via semi-structured email questions asking participants to review MOHR’s content for evidence supporting the tool, feedback on wording, missing content, and content to remove.

### Patient Engagement

A convenience sample of seventeen patients (41% female) was recruited from two primary care practices, in Richmond and Fairfax, Virginia. Clinicians identified patients with MCCs who might benefit from using a tool like MOHR. In-person user experience observations and semi-structured interviews were conducted with each patient. Patients navigated MOHR and were prompted for their reactions to the health risk assessment, deciding which topic(s) to address with a care plan, picking a personal goal, selecting strategies, and navigating the system. Patient interactions were audio-recorded and transcribed.

### Analysis

Transcripts and written feedback from stakeholder engagement activities were subjected to qualitative content analysis using template and emergent coding processes [13]. Template-based codes were derived from the literature on health behavior change, user experience, and health information technology. Themes related to the patient-centered care planning processes; digital interface design, usability, and functionality; clinical content accuracy and coherence; and implementation feasibility. We used Microsoft Excel to organize, store, and code the qualitative data. Three authors (KO, AH, HS) coded independently and then met to review themes and resolve discrepancies.

## Results

The four stakeholder groups brought unique perspectives to MOHR’s key features for care plan development, as summarized in Table 2. Both community members and patients highlighted the importance of manageable goals and ongoing support. Community members also focused on the patient navigator relationship and need for accurate and reliable community referrals. Patients recommended patient health education and access to care plan examples. Academics and CSPs both suggested using action-oriented language. Academics prioritized recommendations with an evidence base, while CSPs focused on recommendations with anecdotal support from clinical experience.

**Table 2.** Key stakeholder feedback informing key features of the care planning process and MOHR design

	Community members	Academic researchers	Community care providers	Patients
Risk assessment	• Make initial interactions with the tool relatable by providing health education that is tailored to patients’ specific diagnoses	• Ensure that the health risk assessment questions were evidence-based		• Use the health screener as an opportunity to educate patients and pique their interest
	• *“The disease alone can be overwhelming and then add the extra layers. How do I deal with the diagnosis if all the other things arenʼt working?”*			• *“I think they’re all beneficial to me. I feel like…it’s good for you to know these things so you can figure them out. I kinda wish I had them earlier.”*
Goal creation	• Prompt patients to think about and articulate their source of motivation to make changes • Make sure goals are realistic and manageable for patients	• Make health goals meaningful to patients	• Make sure that goals are not too restricting, to allow for patients to stay motivated	• Incorporate incremental goals to build patient confidence
	• *“If someone experienced homelessness, [some goals] may not feel accomplishable. Donʼt want to ask without tools to help them.”*	• *Ensure patient goals incorporate a quality of life reason behind them*	• *Patients working on nutrition might want to have a piece of fried chicken on Friday or a donut on Sunday*	• *“It’s difficult to move forward if you if the goal is too difficult to do, it’s gotta be manageable small incremental steps. You gotta walk before you run, and it builds confidence when you do. You got a couple of small goals done. You get more confident.”*
Action strategies	• Use language that is simple and easily understood by patients • Provide many options for strategies so patients can identify which ones will best support their goal and are feasible for them	• Use jargon-free and specific language • Phrase strategies positively and proactively • Add strategies with known research support • Prioritize the most feasible and affordable strategies first	• Use jargon-free and specific language • Phrase strategies positively and proactively • Add strategies that are practical and have anecdotal support • Include strategies that are accessible to diverse patient populations	• Use language that is simple and easily understood by patients • Provide multiple options for how to achieve a goal
	• *“Some people may know what [goal] they need but not know how to get it”*	• *For example, change “Drink less caffeine” to “Stop drinking caffeine after 3 PM”*	• *For example, revise "Drink less soda" to "Drink more water"* • *Add strategies such as "Consider buying a noise machine or using a noise machine app"*	• *“Some people, they donʼt understand certain things. You know, simple, simple words is something that everybody could read.”* • *“I would be modifying this list… I do not want to talk to my doctor about counseling so I do not want that on the list, do not really want to go to group counseling, but I could join a peer support group.”*
Patient navigator support	• Make sure navigators check in regularly • Provide patients with support navigating the healthcare system • Navigators must ensure the patient is eligible for community resources and referral information is accurate	• Refer patients to community resources that are both geographically and financially accessible to them	• Provided specific recommendations of community organizations whom they had previous positive experiences referring patients to	• Make sure navigators provide continuous support and show genuine care for the patient
	• *“Relationship with the patient is in jeopardy because the advocate didnʼt give me a resource that worked… broke trust.”*			• *“A prerequisite is a bond…your physician or someone and probably they took the time to at least have the discussion with you.. Just the actual interaction we had was important.”*

Three groups reacted to MOHR’s risk assessment component. Community members wanted the health risk assessment process to educate patients on the connection between health condition management and behavioral, emotional, and social factors. Similarly, patients were often surprised by the relation between risk factors and their physical health. They expressed appreciation for learning how these factors influenced their health, with one patient stating, “I kinda wish I had this earlier.” Finally, the academic researchers focused on ensuring that the questions included in the health risk assessment were evidence-based.

All groups commented on goal creation in MOHR. First, community members suggested clarifying personal sources of motivation and anticipated benefits. Community members and patients discussed the need for ensuring that goals are realistic, voicing concern that patients with unattainable goals may become discouraged. Academic researchers suggested that patients consider ways to meaningfully improve their quality of life when creating goals, in order to enhance patient motivation and commitment. Specifically, tying the goal to a desired outcome, such as, “complete daily exercises to build strength and be able to play with my grandchildren.”

Community care providers cautioned against making goals that are too restrictive, suggesting that incorporating flexibility would keep patients engaged. For instance, a patient working on nutrition may want to eat a “treat” every so often. Patients suggested starting with their smaller goals to allow for patients to build self-efficacy before addressing larger goals.

All four groups provided recommendations for MOHR action strategies. Each group recommended removing jargon and improving language specificity. This was not limited to medical terminology, as they expressed a general preference for using words accessible to those at lower reading levels. They identified phrases to improve specificity, such as revising “improve sleep environment” to include specific strategies, such as the use of sound machines or sleep-aid apps. Both academic and CSP participants preferred strategies framed positively and proactively. For example, they suggested replacing “stop eating unhealthy snacks” with “replace processed snacks with those that are high in protein, low-fat, or fresh.” CSP participants recommended new strategies with anecdotal support, regardless of research support. For example, they suggested adding strategies such as “sleep with a white noise machine.” Conversely, academics suggested adding only strategies with research support. Academic researchers also stated the tool’s organization of action strategies was paramount for patient comprehension of the topics. They proposed headers for each section of patient strategies to enhance quick understanding of the information. Community members and patients wanted multiple examples of action strategies to choose from while creating their care plan to pick ones best suited for their lifestyle and goals.

All four groups had suggestions related to the patient navigator role which supports patients throughout the care planning process. Community members and patients indicated that providing ongoing support outside of the tool was necessary, highlighting the importance of the patient navigator relationship. They preferred that patient navigators initiate regular check-ins and be easily accessible for ad hoc support, when needed. Patient participants suggested including contact information for patient navigators in the tool so that patients can reach out for help directly. Community members also recommended verifying resources before making a referral to community resources, reporting that referrals pose risk of damaging patient trust when referral information is inaccurate or contacts are difficult to reach. Academics identified potential patient barriers with community resources, advising patient navigators to provide referrals that are geographically and financially accessible. CSP participants provided specific recommendations of “trusted” community organizations they had past positive experiences with.

## Discussion

This report summarizes an efficient procedure for eliciting important input from four key stakeholder groups and how it informed the development of a web-based care planning process and platform. Across groups, there were both unique and shared recommendations, that together should improve the functionality and use of our tool and support program. Recommendations ranged from simple language alterations that made the tool more patient-friendly to describing meaningful ways for patient navigators to support patients and to promote patient care planning success.

Health services research and the healthcare industry commonly seek user feedback to improve research aims or products. Health risk screeners and decision aid tools must undergo rigorous validity and user testing, which relies on clinician, patient, and public engagement [14,15]. Patient and public engagement has also been used for developing health IT, such as health screening web pages, mobile health records, and patient information sharing platforms [6,16]. Meaningful engagement throughout development results in broadening the “reach” of a product, by ensuring that patients are better able to use and navigate it. Engaging patients, clinicians, or a community has been shown to improve patient experiences and desired outcomes. However, the singular engagement of each group is insufficient on its own. Our findings highlight the benefits of blending perspectives from multiple groups and how they contributed to the development of a more practical, actionable, and helpful care planning tool.

A limitation of this study is the timing and method of feedback. Feedback from each group was solicited at different stages of development. The stage of development was, importantly, pertinent for the domain of feedback they provided (i.e., patient feedback was not sought until after the tool had been developed in a mature, usable form rather than conceptual.) We also solicited feedback using various methods across groups; the type of feedback method was purposefully selected to promote what we considered the most thoughtful feedback from each group.

## Conclusion

Our approach with multiple stakeholder engagement offers insight for researchers and healthcare providers designing similar interventions. It is imperative to solicit and incorporate feedback from a range of stakeholders to develop interventions that are more practical, actionable, and helpful. As demonstrated here, the participation of community members, researchers, community care providers, and patients throughout development provided important complementary perspectives to develop a robust care planning tool.

## References

[1] Glasgow RE , Davis CL , Funnell MM , Beck A. Implementing practical interventions to support chronic illness self-management. The Joint Commission Journal on Quality and Safety 2003; 29(11): 563–574. DOI 10.1016/s1549-3741(03)29067-5. 14619349

[2] Miller KL. Patient centered care: a path to better health outcomes through engagement and activation. NeuroRehabilitation 2016; 39(4): 465–470. DOI 10.3233/NRE-161378. 27689606

[3] Khalifehzadeh-Esfahani A , Amirzadeh A , Golshahi J. Effect of a care plan on the quality of life of the patients with atrial fibrillation. Iranian Journal of Nursing and Midwifery Research 2018; 23(4): 277–280. DOI 10.4103/ijnmr.IJNMR_35_16. 30034487 PMC6034522

[4] Mikkola I , Hagnäs M , Hartsenko J , Kaila M , Winell K. A personalized care plan is positively associated with better clinical outcomes in the care of patients with type 2 diabetes: a cross-sectional real-life study. Canadian Journal of Diabetes 2020; 44(2): 133–138. DOI 10.1016/j.jcjd.2019.05.003. 31399365

[5] Glasgow RE , Huebschmann AG , Krist AH , Degruy FV. An adaptive, contextual, technology-aided support (ACTS) system for chronic illness self-management. The Milbank Quarterly 2019; 97(3): 669–691. DOI 10.1111/1468-0009.12412. 31424137 PMC6739607

[6] Krist AH , Glenn BA , Glasgow RE , et al. Designing a valid randomized pragmatic primary care implementation trial: the my own health report (MOHR) project. Implementation Science 2013; 8(1): 73. DOI 10.1186/1748-5908-8-73. 23799943 PMC3694031

[7] Kilbourne AM , Goodrich DE , Miake-Lye I , Braganza MZ , Bowersox NW. Quality enhancement research initiative implementation roadmap: toward sustainability of evidence-based practices in a learning health system. Medical Care 2019; 57(Suppl 3): S286–S293. DOI 10.1097/MLR.0000000000001144. 31517801 PMC6750196

[8] Moullin JC , Dickson KS , Stadnick NA , Rabin B , Aarons GA. Systematic review of the exploration, preparation, implementation, sustainment (EPIS) framework. Implementation Science 2019; 14(1): 1. DOI 10.1186/s13012-018-0842-6. 30611302 PMC6321673

[9] Woolf SH , Zimmerman E , Haley A , Krist AH. Authentic engagement of patients and communities can transform research, practice, and policy. Health Affairs 2016; 35(4): 590–594. DOI 10.1377/hlthaff.2015.1512. 27044956 PMC4868544

[10] O’Cathain A , Croot L , Duncan E , et al. Guidance on how to develop complex interventions to improve health and healthcare. BMJ Open 2019; 9(8): e029954. DOI 10.1136/bmjopen-2019-029954. PMC6701588 31420394

[11] Majid U , Kim C , Cako A , Gagliardi AR. Engaging stakeholders in the co-development of programs or interventions using Intervention Mapping: a scoping review. PLoS One 2018; 13(12): e0209826. DOI 10.1371/journal.pone.0209826. 30586425 PMC6306258

[12] Zimmerman EB , Haley A , Creighton GC , et al. Assessing the impacts and ripple effects of a community-university partnership. Michigan Journal of Community Service Learning 2019; 25(1): 62–76. DOI 10.3998/mjcsloa.3239521.0025.106. 32905315 PMC7470035

[13] Bernard R , Wutich A , Ryan G. Content analysis. In: Analyzing Qualitative Data. 2nd ed. Los Angeles, CA: Sage, 2017, 242–268.

[14] Akl EA , Grant BJ , Guyatt GH , Montori VM , Schünemann HJ. A decision aid for COPD patients considering inhaled steroid therapy: development and before and after pilot testing. BMC Medical Informatics and Decision Making 2007; 7(1): 12. DOI 10.1186/1472-6947-7-12. 17504536 PMC1877801

[15] Kroenke K , Spitzer RL , Williams JBW. The PHQ-9. Journal of Medical Care 2001; 46202: 606–613. DOI 10.1097/01.MLR.0000093487.78664.3C.

[16] Perfetto EM , Harris J , Mullins CD , dosReis S. Emerging good practices for transforming value assessment: patients’ voices, patients’ values. Value Health 2018; 21(4): 386–393. DOI 10.1016/j.jval.2017.11.013. 29680093

